# Supplementary material for: The effect of COVID-19 vaccination on the menstrual pattern and mental health of the medical students: A mixed-methods study from a low and middle-income country
Source: PLoS One. 2022 Nov 10;17(11):e0277288. doi: 10.1371/journal.pone.0277288 (PMC9648815; doi:10.1371/journal.pone.0277288)
Supplement: Attachment — Submitted filename: Rebuttal letter PONE-D-22-20959 120922.docx [file pone.0277288.s001.docx]

**Rebuttal Letter: Article No: PONE-D-22-20959, titled “The Effect of COVID-19 Vaccination on the Menstrual Pattern and Mental health of the Medical students: A Mixed-Methods Study from a Low and Middle Income Country”**

Dear Editor,

Let me start with thanking the worthy reviewers for appreciating the study and considering it for proceeding further. We have tried to address all the suggestions of the reviewers within the manuscript. Please find point by point response to the reviewers’ comments.

**Comment: There is a need for a stronger rationale for the study.**

***Answer:*** *This has been addressed and highlighted in the manuscript.*

**Comment: Manuscript should meet PLOS ONE style requirement including those for file naming.**

*Answer: This has been addressed as advised.*

**Comment: Please amend either the title on the online submission form or the title in the manuscript, so that they are identical.**

*Answer: The title on the manuscript and the online submission form are same.*

**Comment: Mention about the type of mixed methods study design.**

*Answer: This has been addressed and highlighted in the manuscript*

**Comment: Discussion section does not integrate both results from the qualitative and the quantitative together.**

*Answer: This integration has been addressed in the conclusion, which is technically the part of Discussion section.*

C**omment: What is the number of full-dose vaccines in other countries?**

*Answer: Although the authors were not able to understand this comment exactly. However, as per our understanding of the comment, most of the vaccines in use are having two doses and the number of different vaccines in use are 6, as mentioned in the paper.*

**Comment: Cronbach’s alpha values of the scale used in this research should be added.**

*Answer: Since the values of scales calculated in our study have been mentioned in the paper, the authors did not find it necessary to add the values of the original scales which are .83 and .87 respectively for HADS and MSQ, respectively. However, if the editorial board suggests these to be added, the authors allow the copy editor to make the necessary changes.*

Sincerely Yours,

**Prof Dr Muhammad Irfan (Corresponding Author, on behalf of all authors)**

**MBBS, MCPS** (Psych), **FCPS** (Psych), **CHPE, MS** (Mental Health Policy & Services), **PhD.**

Professor & Head, Department of Mental Health, Psychiatry & Behavioral Sciences, Peshawar Medical College, Peshawar.

Director Research, Prime Foundation Pakistan

President-Elect, World Association of Cultural Psychiatry

Director Research & Innovation, Asian Cognitive Behavioral Therapists Association

President Pakistan Association of Medical Editors
