# Supplementary material for: The effect of COVID-19 vaccination on the menstrual pattern and mental health of the medical students: A mixed-methods study from a low and middle-income country
Source: PLoS One. 2022 Nov 10;17(11):e0277288. doi: 10.1371/journal.pone.0277288 (PMC9648815; doi:10.1371/journal.pone.0277288)
Supplement: Attachment — Submitted filename: Rebuttal letter PONE-D-22-20959 151022.docx [file pone.0277288.s002.docx]

**Rebuttal Letter: Article No: PONE-D-22-20959, titled “The Effect of COVID-19 Vaccination on the Menstrual Pattern and Mental health of the Medical students: A Mixed-Methods Study from a Low and Middle Income Country”**

Dear Editor,

Let me start by thanking the worthy reviewers for appreciating the study and considering it for proceeding further. We have tried to address all the suggestions of the reviewers within the manuscript. Please find point by point response to the reviewers’ comments.

**Comment 1:** Please amend the title either on the online submission form or in your manuscript so that they are identical.

***Answer:*** *Checked. (Line 1& 2, page 1).*

**Comment 2:** Please amend your manuscript to include a reference list. References must be placed at the end of the manuscript and numbered in the order that they appear in the text.

***Answer:*** *The manuscript already has the reference list (Line 475-551, page 21-24).* *They are placed at the end of the manuscript and numbered in the order that they appear in the text.*

Sincerely Yours,

**Prof Dr Muhammad Irfan (Corresponding Author, on behalf of all authors)**

**MBBS, MCPS** (Psych), **FCPS** (Psych), **CHPE, MS** (Mental Health Policy & Services), **PhD.**

Professor & Head, Department of Mental Health, Psychiatry & Behavioral Sciences, Peshawar Medical College, Peshawar.

Director Research, Prime Foundation Pakistan

President-Elect, World Association of Cultural Psychiatry

Director Research & Innovation, Asian Cognitive Behavioral Therapists Association

President Pakistan Association of Medical Editors
